# Sporadic lymphangioleiomyomatosis with lymphocyte-predominant pleural effusion misdiagnosed as tuberculosis: a case report

**DOI:** 10.1016/j.rmcr.2026.102491

**Published:** 2026-08-28

**Authors:** Beniam Tadewos Lamboro, Mariamawit Belachew Dejene, Telila Kumneger Belisa, Mulualem Gashaw Dessie

**Affiliations:** College of Health Sciences, School of Medicine, Addis Ababa University, Addis Ababa, Ethiopia

**Keywords:** Lymphangioleiomyomatosis, Chylothorax, Tuberculosis, Misdiagnosis, Sirolimus, Ethiopia

## Abstract

Lymphangioleiomyomatosis (LAM) is a rare cystic lung disease that primarily affects women of reproductive age. The disease may involve extrapulmonary sites, including the lymphatic system, and can present with a lymphocyte-predominant pleural effusion mimicking tuberculosis. We report a 35-year-old woman who presented with a three-year history of progressive dyspnea and recurrent lymphocyte-predominant exudative pleural effusions. She was empirically started on anti-TB therapy at a local hospital but showed no clinical improvement. Upon evaluation at a tertiary center, there was decreased air entry over the posterior third of the chest. Repeat pleural fluid analysis demonstrated a milky appearance with elevated triglyceride levels, consistent with chylous effusion. High-resolution CT showed diffuse, bilateral, thin-walled pulmonary cysts and bilateral pleural effusion with retroperitoneal lymphatic involvement, which confirmed the diagnosis of LAM. Treatment with sirolimus was started and showed significant clinical improvement, but the patient discontinued therapy after two months due to financial constraints. In this patient, LAM presented as tuberculous pleuritis that delayed the definitive diagnosis. This should alert clinicians in high TB burden settings to consider this rare disease in reproductive-aged women who present with progressive dyspnea and a lymphocyte-predominant pleural effusion, particularly when there is no response to anti-TB therapy.

## Highlights

• LAM can present as lymphocyte-predominant pleural effusion mimicking TB.
• LAM diagnosed with HRCT findings without need for lung biopsy.
• Sirolimus improved symptoms but limited by financial constraints.

## 1 Introduction

Lymphangioleiomyomatosis (LAM) is a rare, progressive cystic lung disease characterized by abnormal proliferation of smooth muscle–like cells [[1], [2], [3]]. It primarily leads to diffuse destruction of lung parenchyma but also affects other organs, including lymphatic system and kidneys [1,2].

The disease occurs either sporadically or in association with tuberous sclerosis complex and predominantly affects women of reproductive age, with an estimated prevalence of 3–8 cases per million [1,2,4]. It is exceedingly rare in men and children [2,4].

Clinically, patients present with cough, progressive dyspnea, and recurrent pneumothorax [2,3]. Chylothorax is also a recognized manifestation, occurring in approximately 10–20% of patients [1,5]. The disease is often underrecognized in regions where respiratory symptoms are frequently attributed to infection [6]. We report a 35-year-old Ethiopian woman initially treated for tuberculosis without improvement, subsequently diagnosed as sporadic LAM complicated by chylous pleural effusion and lymphatic involvement.

## 2 Case presentation

A 35-year-old woman presented with progressive shortness of breath of three years duration. Her symptoms began with exertional dyspnea, which gradually worsened and limited her daily activities. She also experienced a dry cough, pleuritic chest pain, and easy fatigability.

She had regular menses and did not use hormonal contraceptives. She reported no history of fever, weight loss, night sweats, hemoptysis, orthopnea, or paroxysmal nocturnal dyspnea. She denied any known childhood lung disease, connective tissue disease, smoking, significant environmental or animal exposure, or family history of similar illness.

On physical examination, the patient was clinically stable. Her vital signs were within normal range with blood pressure of 110/75 mmHg, pulse rate of 84 beats per minute, respiratory rate of 22 breaths per minute, temperature of 36.4°C, and oxygen saturation of 96% on room air. Upon chest examination there was a decreased air entry over right lower posterior third. All other examinations were unremarkable.

Laboratory investigations, including complete blood count, renal and liver function tests, were within normal range. Erythrocyte sedimentation rate (ESR) was 4 mm/hr, and C-reactive protein (CRP) was <5 mg/L. Sputum GeneXpert testing was negative.

Initial pleural fluid analysis at a peripheral hospital revealed a serous, lymphocyte-predominant exudative effusion. The patient was empirically started on anti-tuberculosis therapy but showed no clinical improvement. Later, repeat pleural fluid analysis at a tertiary center showed a milky appearance with elevated triglyceride levels. Pleural fluid cytology revealed lymphocytic predominance (90%) with no malignant cells.

**Fig. 1:**
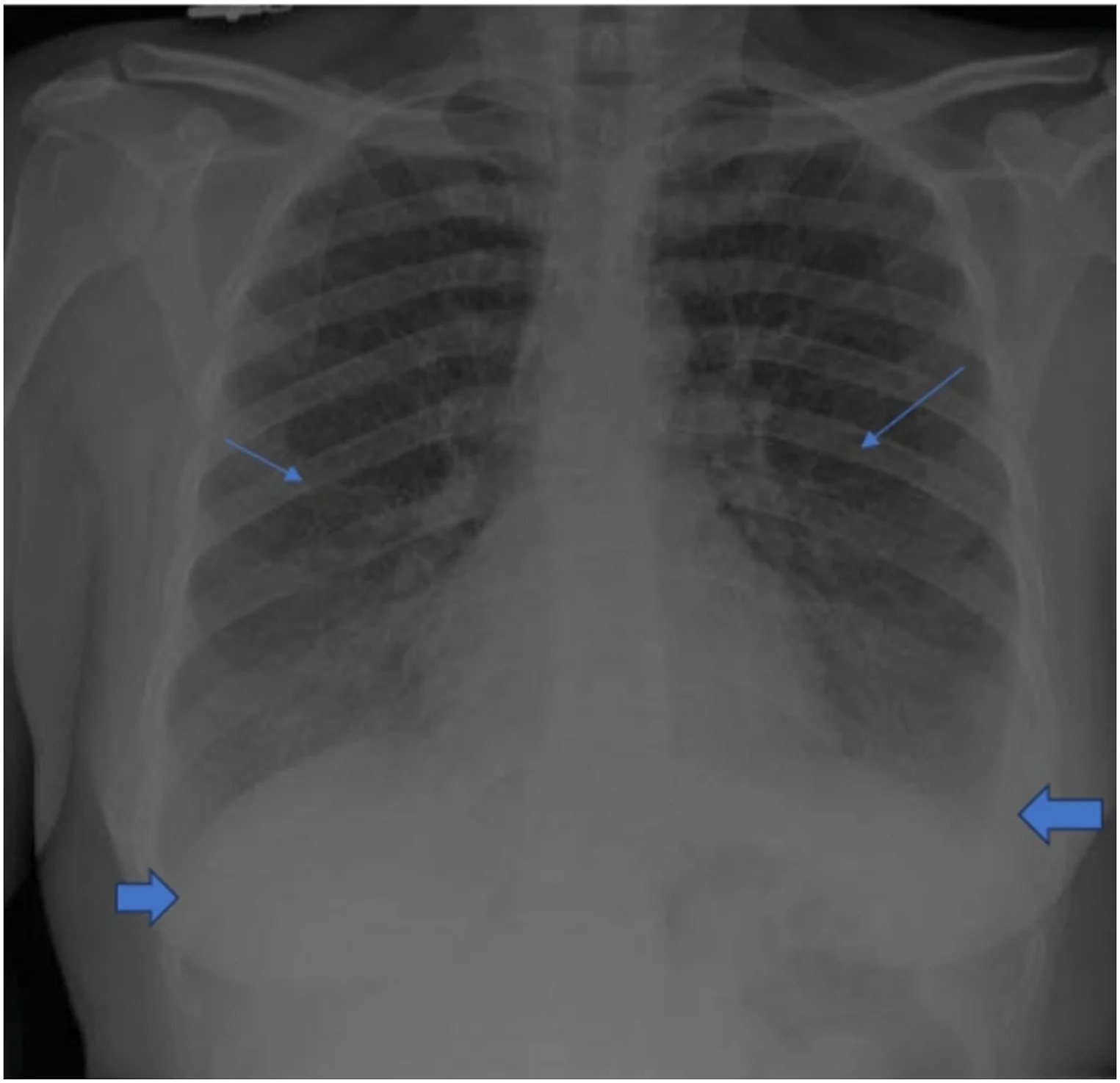
AP chest radiography shows bilateral diffuse reticular pattern (arrows) and bilateral blunted costophrenic angle (block arrows).

**Fig. 2:**
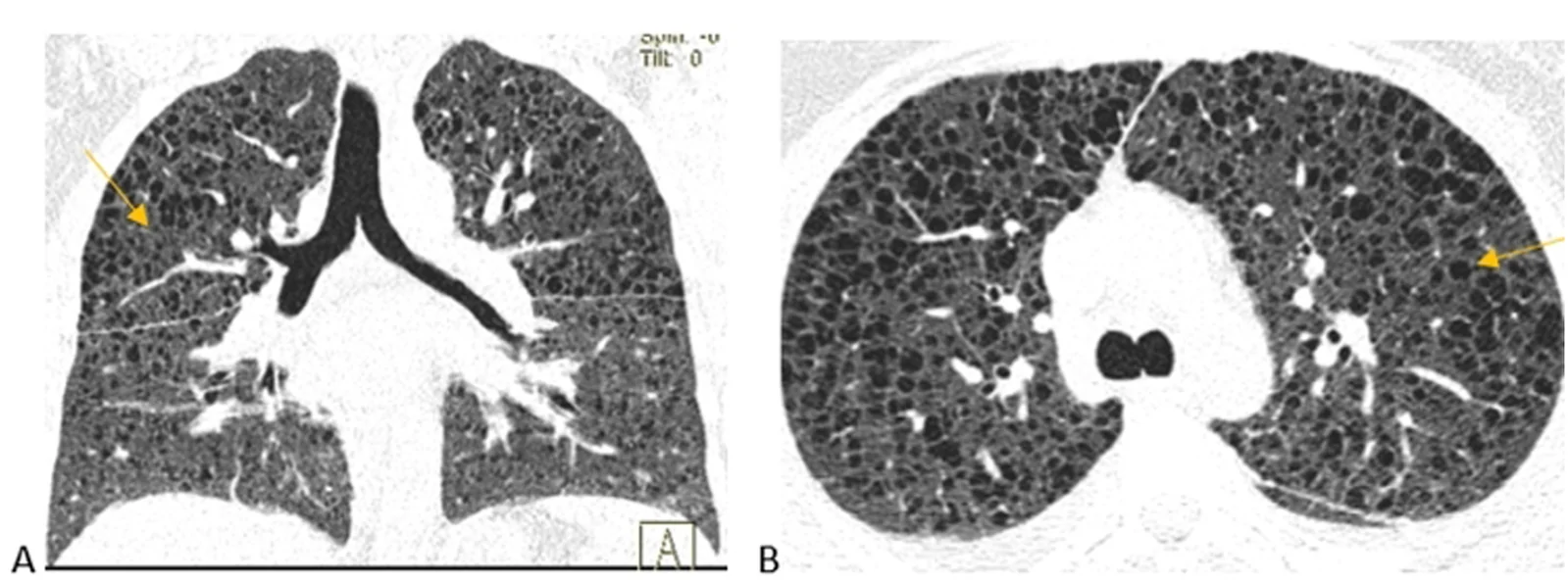
Chest CT lung window coronal (A) and axial (B) plane show bilateral symmetric numerous thin-walled cysts of similar size (arrows) with preserved normal intervening parenchyma.

**Fig. 3:**
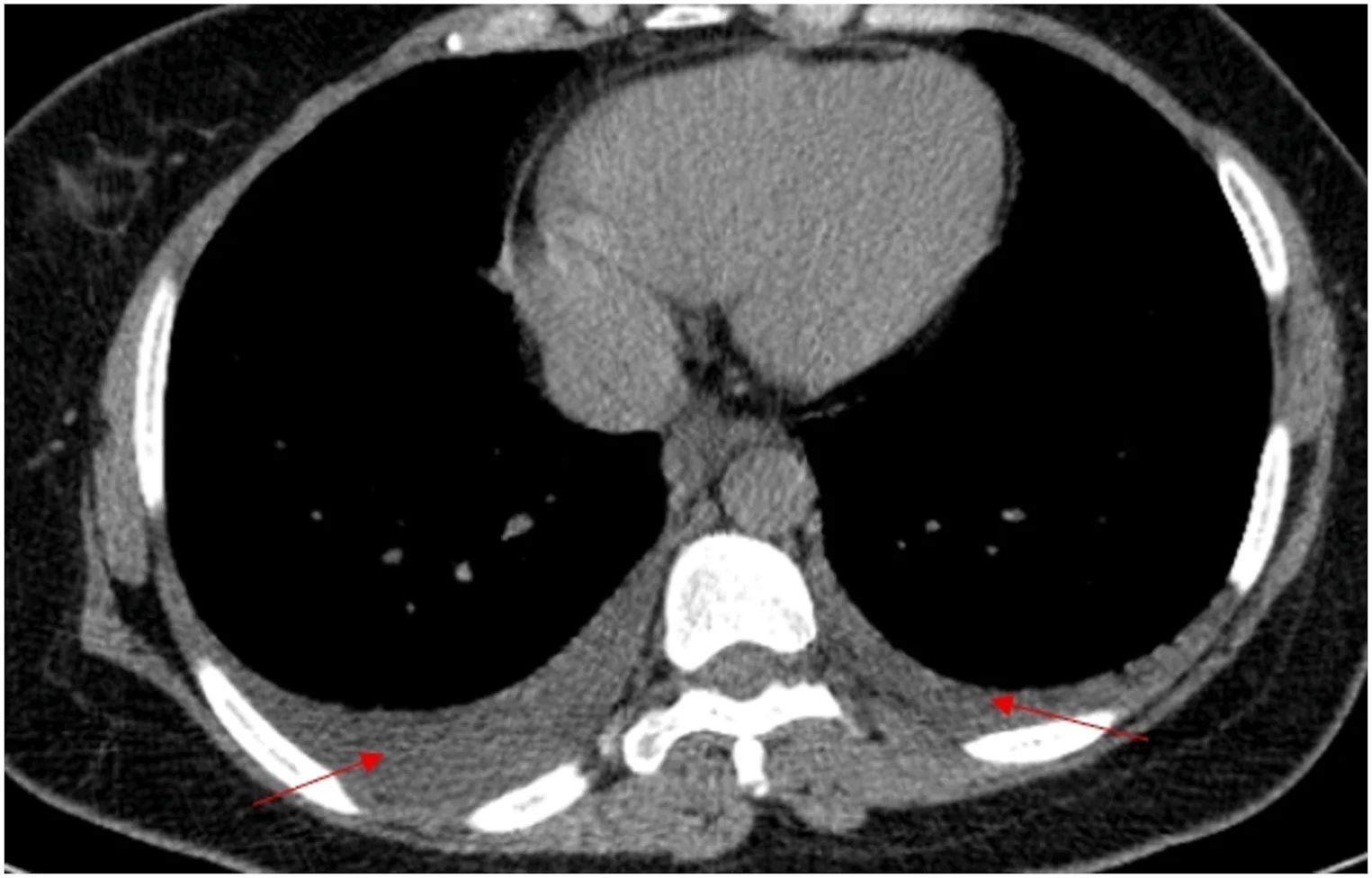
Chest CT soft tissue window axial plane shows bilateral pleural effusion (arrows), with a massive collection on the right side, likely representing chylous effusion.

On chest radiography there were bilateral reticular opacities in the mid- and lower lung zones and blunting of the costophrenic angle (Fig. 1). High-resolution CT demonstrated diffuse, bilateral, thin-walled pulmonary cysts (Fig. 2) and bilateral pleural effusion, with a massive collection on the right side (Fig. 3). Retroperitoneal cystic lesions and associated lymphadenopathy also noticed. Further imaging with echocardiography revealed severe pulmonary hypertension with right atrial dilatation. Serum vascular endothelial growth factor-D (VEGF-D) level was not measured due to limited availability.

Based on the clinical, radiological, and pleural fluid findings, a diagnosis of sporadic LAM was established. The patient was started on sirolimus 2 mg PO daily.

Within two weeks, her dyspnea and exercise tolerance significantly improved. She was able to perform her daily activities. However, she discontinued the medication due to financial constraints.

## 3 Discussion

In high TB burden regions, tuberculosis remains a leading cause of lymphocyte-predominant pleural effusion and is often treated empirically without a definitive diagnosis [7]. Our patient presented with exudative effusion which was interpreted as TB and started on anti-TB therapy. However, relying on clinical suspicion alone can lead to misdiagnosis and inappropriate treatment. Therefore, a systematic approach is essential to differentiate between various etiologies of lymphocyte-predominant exudative effusions [8].

The differential diagnosis of a lymphocyte-predominant exudative pleural effusion is broad and includes tuberculous pleuritis, malignancy, chylothorax, connective tissue diseases such as rheumatoid arthritis and systemic lupus erythematosus, and other rare conditions [8,9]. In high-burden settings, tuberculosis remains the most common cause, but malignancy and chylothorax are also important considerations [7].

Furthermore, chylothorax may not always present with the classic milky appearance which can mislead clinicians [8,10]. In one retrospective study of 74 patients with confirmed chylothorax found that the pleural fluid appeared milky in only 44% of cases [10]. This occurs when patients are fasting or malnourished, as the fat content of chyle decreases significantly without dietary intake, resulting in a serous or slightly cloudy fluid [10]. Similarly, a slow or diluted chyle leak may also fail to produce a milky effusion [8,10]. Therefore, the diagnosis of chylothorax should be confirmed by biochemical analysis demonstrating a pleural fluid triglyceride level >110 mg/dL or the presence of chylomicrons, rather than relying solely on visual appearance [10]. In our patient, the initial pleural fluid did not appear milky, which contributed to the misdiagnosis of tuberculosis.

Given the negative GeneXpert result and lack of clinical response to anti-TB therapy, the diagnosis of tuberculosis was excluded. Similarly, malignancy and lymphoma were also considered; however, pleural fluid cytology revealed no malignant cells. The patient's clinical course and image findings suggested alternative diagnosis of rare lung condition called LAM.

LAM is a rare disease traditionally diagnosed via lung biopsy [[1], [2], [3]]. But in current era, more than 70% of LAM cases are being diagnosed based on diagnostic criteria proposed by the joint American Thoracic Society/Japanese Respiratory Society (ATS/JRS) guidelines [1]. This includes characteristic HRCT findings combined with extrapulmonary features. In our patient, the diagnosis was established based on HRCT findings of diffuse, bilateral, thin-walled cysts combined with chylous pleural effusion. This reduced the need for the invasive procedure [1,2].

In addition to radiological and clinical features, demographic features as a young premenopausal woman also support the diagnosis [11,12]. Our patient also developed retroperitoneal cystic lesions and lymphatic abnormalities. This multisystem involvement is consistent with the known pathophysiology of LAM as a systemic disease [4,5].

At the molecular level, LAM is driven by mutations in the TSC1 or TSC2 genes, which activate mammalian target of rapamycin (mTOR) pathway [1,4]. This promotes abnormal smooth muscle–like cell proliferation and progressive cystic destruction of the lung parenchyma [13,14]. Sirolimus, an mTOR inhibitor, counteracts this process by suppressing dysregulated mTOR signaling, reducing cellular proliferation [14]. Many reviews support that sirolimus improves chylous effusions and stabilizes lung function [1,4,5,11]. Similarly, our patient showed significant clinical improvement after initiation of sirolimus.

However, sirolimus commonly causes mucositis, hyperlipidemia, and increased risk of infections [1,4,5]. These adverse effects are mild and usually do not lead to drug discontinuation [1,2]. Our patient did not experience these side effects; however, discontinued therapy due to financial constraints. This highlights an important treatment challenge in resource-limited settings.

Long-term prognosis of the disease is determined by multiple factors [15]. In our patient, the presence of severe pulmonary hypertension and right atrial dilatation on echocardiography adversely influences clinical outcomes. Furthermore, patients require long-term treatment because the protective effect of mTOR inhibition is reversible and disease progression resumes when the medication is stopped [1,2]. In our case discontinuation of sirolimus places her at substantial risk for disease progression, recurrent chylous effusions, and accelerated decline in lung function [2,15].

In addition, emerging evidence suggests that the disease progresses more rapidly in young, premenopausal patients [16]. However such group of patients showed a significant response to mTOR inhibitor therapy compared to postmenopausal patients [16]. Our patient is a young premenopausal woman and showed a clinical improvement during her brief course of mTOR inhibitor therapy. Therefore her prognosis may be more favorable if treatment is resumed.

## 4 Limitations

This case report has several limitations. First, the diagnosis was established radiologically without histological confirmation, though this approach is consistent with current ATS/JRS guidelines. Second, serum VEGF-D levels, which could have provided additional diagnostic and prognostic information, were not available. Third, pulmonary function testing was not performed due to resource limitations, preventing objective quantification of disease severity and treatment response. Finally, genetic testing for TSC1/TSC2 mutations was not available.

## 5 Conclusion

This case underscores that LAM can present with a lymphocyte-predominant exudative pleural effusion closely mimicking tuberculosis. The three-year diagnostic delay in our patient highlights the importance of including LAM in the differential diagnosis for reproductive-aged women with progressive dyspnea and a pleural effusion that fails to respond to anti-TB therapy. Reassessment with advanced imaging and pleural fluid analysis is essential for timely and accurate diagnosis.

## CRediT authorship contribution statement

**Beniam Tadewos Lamboro:** Writing – review & editing, Writing – original draft, Project administration, Conceptualization. **Mariamawit Belachew Dejene:** Validation, Resources, Data curation. **Telila Kumneger Belisa:** Writing – review & editing, Supervision, Methodology, Formal analysis. **Mulualem Gashaw Dessie:** Validation, Resources, Investigation, Data curation.

## Consent statement

Written informed consent was obtained from the patient for publication of this case report and any accompanying images. A copy of the written consent is available for review by the editor-in-chief of this journal.

## Ethics approval

Ethics approval was not required for this single case report at our institution. All procedures were performed in accordance with institutional guidelines.

## Data availability statement

All data relevant to this case report are included in the manuscript. Anonymized patient data are available from the corresponding author upon reasonable request.

## Clinical trial number

Not applicable.

## Use of AI assisted technologies

During the preparation of this work, the authors used Grammarly and ChatGPT (OpenAI) for language polishing and grammar correction. The authors reviewed and edited the output as needed and take full responsibility for the content of the published article.

## Funding

No funding was received for this work.

## Declaration of competing interests

The authors declare that they have no known competing financial interests or personal relationships that could have appeared to influence the work reported in this paper.

## Abbreviations

LAM –: Lymphangioleiomyomatosis
TB –: Tuberculosis
HRCT –: High-Resolution Computed Tomography
ATS –: American Thoracic Society
JRS –: Japanese Respiratory Society
TSC –: Tuberous Sclerosis Complex
mTOR –: Mammalian Target of Rapamycin
VEGF-D –: Vascular Endothelial Growth Factor-D
ESR –: Erythrocyte Sedimentation Rate
CRP –: C-Reactive Protein
